# Small-Pox Considered with Particular Reference to Morbid Anatomy, and More Especially to the Occurrence of Pocks on Internal Parts

**Published:** 1838-04

**Authors:** 


### Art. XI.

*Die Pocken-krankheit mit besonderer Rücksicht auf die Pathologische Anatomie. (Es giebt Pocken auf inneren Theilen.)* Von Alexander Petzholdt, &c. *Mit 4 Tafeln abbildungen.—Leipzig*, 1836. 4to. pp. 120.

*Small-Pox considered with particular Reference to Morbid Anatomy, and more especially to the occurrence of Pocks on internal Parts.* By Dr. Alexander Petzholdt.—*Leipzig*, 1836.

In the winter of 1832-33, small-pox prevailed epidemically at Leipzig, occasioning considerable mortality. The author of the monograph before us, then in attendance as a student at St. Jacob's Hospital, under Professor Clarus, had an opportunity of performing, and of seeing performed, both in that institution and in private, a considerable number of dissections of persons who died of this disease. Since that time he has had further opportunities of extending his researches during the prevalence of an epidemic, though of less severity, at Dresden, where he is now settled. Altogether, he estimates the number of bodies of persons dying of small-pox, which he has had an opportunity of inspecting, as not falling short of forty. In 1833 he published, at Leipzig, his inaugural dissertation, entitled "Observationes quædam de Variolarum cum externarum tum internarum natura." The present more extended treatise is divided into four sections: in the *first*, he treats of the etymology, definition, and antiquity of small-pox; of the form of the pock, the course of the disease, and its consequences: in the *second*, of the morbid appearances found on examination after death: in the *third*, of the etiology, character, diagnosis, and prognosis; and in the *fourth*, of the treatment. Though he seems to have handled all these topics with great industry and ability, it is with the matter contained in the second section that we have been most interested; and to it we purpose at present to direct the attention of our readers.

Dr. Petzholdt has prefixed to his account of the morbid conditions of the Skin, and of the Mucous and Serous Membranes, some remarks on the healthy anatomy of these several textures; of the scope of which our readers may judge from his own summary of the topics on which he principally insists:—1st, that there exists a great correspondence between the structure of the mucous membranes and of the external skin; 2d, that the cuticle, although no nerves or vessels can be perceived in it, is still to be considered as living; 3d, that the cutis is composed of three layers, but these are only physiologically separable, being constituted by the mode in which the blood-vessels are distributed; 4th, that the middle layer is the especial seat of the cutaneous glands; 5th, that these glands are distributed over the whole surface of the body, excepting the palm of the hand and sole of the foot; 6th, that the roots of the hair are not always imbedded in the glands; 7th, that mucous membrane has everywhere an epithelium; 8th, that the chemical difference between cutis and mucous membrane is scarcely appreciable.

The following is the description which Dr. P. has given of the appearances observable on the anatomical examination of the common integuments in small-pox.

*The Epidermis.** If this membrane be examined when the eruption of small-pox first breaks out, its undermost layers are found to be softened, almost spongy, and as if filled with a fluid. If a circular incision be made into the skin round the circumference of a papula, this, being loosened by the cut from its lateral connexion with the skin, can be removed pretty easily with the pincers, in the form of a little knot. This experiment shews that, at the period of the disease in question, the connexion of the cuticle with the cutis is nearly destroyed at those parts of the skin which are affected, whilst a perpendicular section affords us a ready opportunity of satisfying ourselves that there is no cavity beneath the cuticle. During the growth of the pustules, the spongy softening of the cuticle is increased; a still greater quantity of fluid collects between the substance of its lowest layers; there at length arises a small cavity filled with fluid, and, by the increased accumulation of this fluid, the cuticle is gradually pushed upwards. Its elevation is necessarily attended with expansion; the spongy texture is consequently compressed by the fluid, first at the apex of the vesicle, and it afterwards gives way towards its sides, and thus there remain only the superficial layers of the epidermis, which are clear and transparent. Whilst this is the state of parts internally, the pocks appear externally as clear vesicles. In all the pocks in which there exists a pit or umbilical depression, the cuticle is at this period connected with the cutis by a thread, (afterwards to be more particularly considered,) of which scarcely any trace can be found at the period of suppuration. When the pustule is perfectly formed, and therefore quite filled with pus, it is enclosed in only a very thin layer of cuticle; which, however, varies in thickness, as can best be seen by drawing off the cuticle carefully from the pustules, and after removing the purulent matter attached to it by washing it with water, laying it upon a black body, when it appears almost quite black in the centre, shewing that at that part it is very thin and transparent, whilst at the circumference, on the contrary, it is lighter or quite white, and consequently less transparent, or perfectly opaque, from being thicker at the circumference than at the centre. It is also particularly to be remarked that frequently, when we detach the thin covering of a pock, in which there exists a pit or umbilicus, a little hole is found in the centre of the exposed surface, which can never be observed externally, whether we examine the pocks in their natural condition, with the naked eye or with the microscope.

* Under this term the author includes the cuticle, properly so called, and the rete mucosum.

*The Cutis.* When the thin covering of a part of the skin occupied by a pock is removed, the cutis does not come immediately into view, but it is covered by a substance, varying in colour and consistence according to the degree of ripening of the pustule. At the time at which the formation of the cavity or hole described in the preceding paragraph commences, the fluid that covers the cutis is clear; at a later period it is turbid, more tenacious, and at length it becomes pure pus. If all these matters be removed, which is best done by a pretty strong stream of water from a small syringe, so as not to injure any of the subjacent parts, the following appearances can be seen with the aid of a microscope:—In all the pocks, where pus has formed, there remains some of it behind, which cannot be washed off; and, if we employ for these investigations portions of skin that have had their vessels filled with red colouring matter, it can be seen with the naked eye that the pus is, as it were, wedged in between the bundles of vessels, and is retained by them and between them. If an accurate examination be made of the bottom of the pock, which seems sometimes a little raised, on account of the uncommon accumulation of minute vessels, but sometimes a little depressed and as it were eroded, in almost all instances there comes into view a small depression or aperture. Sometimes, indeed, there are several such apertures, according to the condition and varying size of the pustule; but these lie scattered without order or symmetry. In a considerable number of pocks no such openings are to be found, never on the palm of the hand, and seldom on the sole of the foot. Dr. P. never failed to find them in pocks in which there exists a pit. That these apertures belong to the excretory ducts of the cutaneous glands is easily shewn by a perpendicular section, which brings the subjacent glands into view. The portion of the cutis that is not covered with pocks is also in a morbid condition, presenting everywhere a white puriform matter, which, just as in the pustules, adheres firmly to it, and wedges itself in between the plexus of vessels. If the vessels distributed on the upper surface of the cutis be subjected to minute examination in small-pox, which may be best done in skin artificially injected with colouring matter, a remarkable difference is found between those which lie immediately below the pocks, and consequently on their base, and those which surround their borders; for, whilst those which occupy the bases appear sunk in the substance of the cutis, and are not much filled with the colouring matter, those round their circumference project out of the substance of the cutis, and surround the seat of the pock in a radiated manner and in great abundance. Those, again, in the part of the skin remote from the pock, as can be well observed in the discrete form of small-pox, are far from reddening to such a degree the surface of the cutis; and, indeed, have often the appearance as if they lay deeper buried and in smaller numbers in the substance of the skin, and were only seen shining through it. These appearances may, Dr. P. thinks, be very well explained by admitting, with Gendrin, that, in inflammation, the smallest vessels are rendered altogether or partially impervious; whilst in congestion, on the contrary, they are dilated. There unquestionably exists the highest degree of inflammation at the base of the pock; and hence it is that the vessels in this situation are more difficult to fill with injection, whilst those on the circumference of the pock, having been during life more in the state of congestion, become much filled with the injected matter and very prominent. Lastly, Dr. P. remarks that the so-called wind-pocks are by no means empty, but contain on their base, sometimes a dry matter that crumbles between the fingers, and sometimes, and indeed more frequently, a viscid soft mass: it appears as if only the more solid constituents of the puriform matter filling these pocks had remained, whilst the more fluid had disappeared.

*The Cutaneous Glands and Hairs.* The examination of the skin in a person who has died of small-pox brings to light the existence of cutaneous glands, even in those parts of it where, in the healthy state, they cannot easily be detected: for they are all more or less swollen, so as to assume a pyriform shape; and even their excretory ducts are frequently much distended by the secretion of the glands. If a pock be seated exactly on the spot where the excretory duct of a gland opens, the duct is raised upwards with the cuticle. In consequence of the stretching it thus experiences, it at length tears, and is destroyed by the pus in which it floats; so that, when suppuration has made a certain degree of advancement, the duct can no longer be found. But as, at an early period of its stretching, it still possesses some degree of firmness, it draws the part of the cuticle with which it is connected towards itself; and thus the pit or umbilicus is produced. Accordingly, as soon as the excretory duct has either been torn, in consequence of excessive stretching, or been destroyed by suppuration, the umbilicus disappears. There then remains upon the basis of the pock in the cutis, at this part, the above-described punctiform aperture, which is the passage of the excretory duct to the gland, and which can be particularly well seen if the portion of the skin to be examined has been kept for some time in spirit of wine; when these openings gape, in consequence of the contraction of the cutis. The destruction of the excretory duct is commonly accompanied with the falling out of the hair. Provided the whole gland has not been destroyed, the excretory duct, as well as the hair, may grow again: but, when this has happened, as Dr. P. has remarked, in such glands as were seated very superficially on the cutis, a new formation of gland and hair is impossible. The glands appear much more vascular than in their healthy state, which may account for their being commonly so full and so much swollen; for the mass of blood pressing towards the skin in general will excite the glands to the more powerful exercise of their function, and consequently to an increased secretion of sebaceous matter.

As regards the *Fila sudorifera*, Dr. P. professes himself unable to determine whether or no they undergo any morbid alteration in small-pox. At the time when he had ample opportunities of examining fresh portions of small-pox skin, the threads going from the cutis to the cuticle, to which the name of *fila elastica* was given, were usually regarded as the means of union between the cutis and the cuticle; and appeared of too little importance to deserve much time or trouble being expended on them. Now that attention has been turned to their real importance by Purkinje and Wendt, Dr. P. has had recourse to preserved portions of skin taken from small-pox patients, with a view to their examination, and has compared these with fresh skin in a healthy state. The only difference which he has been able to detect is, that the *fila* of the diseased skin appeared larger than those of the healthy: but whether this might have been the case in the fresh state, and what changes they might have undergone in course of time by steeping in spirit of wine, are points respecting which his investigations lead to no conclusion.

We come next to the results which Dr. P. obtained from the examination of the internal parts of the body, in persons dying of small-pox. It is well known to be a subject about which much diversity of opinion has been entertained, whether the formation of small-pox pustules on internal parts does or does not occur at all; and, if it does, on what surfaces or in what organs? Dr. P. has given an exceedingly good summary of the results which former observers have stated themselves to have obtained from their investigations. Our space will not allow of our transferring this to our pages, as we could have wished to do; and we must therefore content ourselves with pointing out, as the principal sources of fallacy against which pathologists, even of the present day, require to be on their guard in judging of this matter, the liability of the glands that furnish the secretion by which mucous surfaces are lubricated to become enlarged in febrile and other disease, and subsequently to ulcerate, exhibiting, during the several stages of their progress, considerable resemblance to small-pox pustules. The fallacies into which some of the older anatomists seem to have fallen,—such as mistaking for small-pox pustules the little collections of muco-purulent matter that appear at the divided extremities of the bronchial ramifications, when in a state of inflammation, or possibly tubercles in parenchymatous organs and serous surfaces, would hardly mislead any competent pathological enquirer of the present day.

*The Cavity of the Mouth and the Tongue.* Sometimes, at the commencement of the disease, small vesicles are visible on the inner parts of the lips and cheeks, as well as on the point of the tongue; but it is impossible to trace the further development of these in the living subject, in consequence of their becoming covered with tenacious mucus. Dr. P. was frequently able, however, to continue his observations relative to them on the dead body, and from these he has deduced the following account of their course.

There appear, on the lips and inner sides of the cheeks, small white spots, of a round or oval form, the centre of which is very frequently somewhat darker in colour. The epithelium is at these places much softened, and at length rises so as to form a small white vesicle, which is at no period transparent, the softened epithelium remaining always opaque and white; it is incapable of any great expansion by the fluid collecting beneath it, and soon bursts. The subjacent mucous membrane is seen to be at some points eroded on its surface. The course of such a pock is consequently very brief; the constantly moist state of the mouth rendering its actual filling with pus and desiccation with the formation of a scab, altogether impossible.

The accurate investigation of the appearances which the tongue presents, is a matter of somewhat greater difficulty. It is well known that, in consequence of the papillæ on its surface, this organ is very uneven, and the epithelium, which surrounds the individual projections very closely, forms such an immense number of very small folds, that it is impossible, in the human subject, at least in the healthy state, to draw it off as a continuous membrane. But, if small-pox matter be effused on the surface of the tongue, then, at the place where a pock is to arise, the epithelium being pushed off from the roots of the papillæ and pressed upwards to their apices, becomes loose, and appears to the eye as a white speck, in the centre of which also there is very frequently found a dark-coloured small circle or point. This circle or point, however, can be seen for only a very short time, as the whole of the raised part of the epithelium very soon appears white; and this also soon disappears entirely in the living subject, the tongue commonly becoming covered with a thick and variously coloured mucus. If the tongue be examined in the dead body, particularly after it has been subjected for some days to maceration, it is found that the coating of mucus, which is frequently several lines in thickness, can be very easily removed with the handle of the scalpel; the epithelium, which cannot be distinguished from mucus, and which indeed appears nearly dissolved into mucus, being removed at the same time. But, on the apex of each lingual papilla, there remains a trace of the epithelium, and giving to the papilla the appearance of a small mushroom, with its top adhering to the circumference of the stalk. On the sides, on the contrary, the papillæ are bare, and deprived of epithelium; as is best seen by subjecting some of them to the microscope. At the point where, at an earlier stage of the disease, there were seen in the living subject those white and somewhat raised spots that have been mentioned, there is found a depression of the mucous membrane, of a round or of an oblong or oval shape; in which last cases examination shews that the depression is formed by the coalescence of two contiguous pits. On a first inspection of such a depression, the suspicion readily occurs that it is formed by the loss or destruction of some papillæ; but Dr. P., having repeatedly had an opportunity to observe such depressions at their commencement, has satisfied himself that they are not produced by the destruction of papillæ, but by the papillæ being pressed aside by a fluid secreted between their roots, and accumulating there. That it is only in this way that these seeming holes in the tongue are produced, is also shewn by this circumstance, that the papillæ which form the sides of such a depression converge towards its basis, and consequently their apices only are separated from one another. Dr. P. is far from thinking, however, that the papillæ adjoining such a depression may not, sooner or later, be destroyed by suppuration; but that the commencement of these depressions is produced in the manner that has been described, without any destruction of their substance. Those papillæ which are in the immediate neighbourhood of such a depression, and which therefore form its walls and its margin, are somewhat enlarged, and are covered on their apices by a greater accumulation of mucus than the others. In the base of the pit itself, Dr. P. found, though not invariably, the excretory duct of a gland; and the mucous membranes frequently exhibited erosions of various depths. As to the mucous glands or follicles of the tongue, it is well known that they are so closely set on the back part of this organ as to make it appear almost as if covered with them. Dr. P. found them almost always very much distended, and with widely gaping mouths. This, however, is still more the case in the tonsils and in the mucous glands which are seated in the back part of the cavity of the mouth, in the soft and the hard palate, and in the uvula; in which situations one can, without trouble, introduce a large sound into the open glands. In this state of the glands it can be very clearly seen how they are formed only out of folds or pouches of the mucous membrane. All of them abound in thick tenacious mucus. Dr. P. has never found pocks on any of these parts.

*The Pharynx.* Here also Dr. P. was unable, in frequent examinations, to discover small-pox pustules, or any trace of them; but the glands, particularly on the upper part of the pharynx, shewed the same fulness and gaping as those in the back part of the cavity of the mouth. They frequently bore a resemblance to a pock of the outer skin, flattened on its apex, so that an inattentive examiner might easily mistake them for such: but their true nature was fully established by their rounded openings, which could not be torn at any point; by their being obviously lined with mucous membrane; and by the principal orifice of the gland being often perceptibly connected with several other neighbouring orifices. The epithelium over the whole surface of the pharynx was thicker than usual, and could easily be stripped off in patches.

*The Œsophagus.* This portion of the alimentary canal Dr. P. found, in several instances, studded with true pocks. As on the external skin, so also in the œsophagus, a fluid is secreted at a number of points on the surface of the mucous membrane, which detaches and raises the epithelium, and thus forms a pustule. The epithelium is in this way commonly very loose, and softer and thicker than in healthy subjects, and cannot undergo any considerable degree of elevation and distension without tearing. The pustule consequently flattens at a very early period, and discharges the fluid which it contains, so that it is very rare to meet with one that is unbroken; in place of which there is usually found only an abscess partially covered by the raised and loosened epithelium. It is obvious, therefore, that the perforation of the epithelium, produced by the bursting of the pustules, cannot have any determinate form; and accordingly the author found them to be sometimes round, sometimes triangular or quadrangular, or to consist of a mere fissure. If the epithelium be removed, as can easily be done, there can be seen upon the proper mucous membrane eroded portions, which correspond with the perforations and fissures in the epithelium, but are sometimes of greater circumference than one would presume from the external apertures: at other places, however, the mucous membrane is quite free from erosion, and, when the epithelium has been drawn off, all that is to be seen is an effused matter. These erosions, when minutely examined, Dr. P. always found to be round or oval; and he never saw them so deep as to have perforated the true mucous membrane. On the contrary, the *membrana vasculosa*, or cellular texture connecting the mucous with the muscular coat, was sound and uninjured. On the base of many of the pocks there was found a white body, which Dr. P. ascertained to be a gland. The glands of the œsophagus, according to his observations, are seated, for the most part, below the proper mucous membrane; but as in the bodies of persons dying of the small-pox, which he examined, these glands were greatly distended with mucus, they could easily be seen through the thin proper mucous coat, particularly where it was eroded, and had the appearance as if they actually lay in the bottom of the small ulcer. But, when the mucous membrane was carefully removed with a knife, the glands remained seated in the membrana vasculosa; whilst the mucous coat, thus stripped off, was seen to be unperforated; which could not have been the case if the glands were actually imbedded in it. Dr. P. very seldom found such a gland affected, when it lay accidentally in the true mucous coat. It appears not infrequently to happen that the epithelium of the œsophagus is quite destroyed, and as if completely converted into mucus, coming away, in washing the surface, along with the mucus which covers it.

*The Stomach and Intestinal Canal.* In the stomach Dr. P. found nothing that could be conceived to depend on small-pox, except very great enlargement of the glands and slight softening of the mucous membrane: but, in the rest of the intestinal canal, particularly in the small intestine, his attention having been several times called to portions of different sizes, and not of any determinate or regular form, by the circumstance of their not glistening in the light, like the surrounding mucous membrane, he found, on accurate examination with a simple microscope, that the papillæ of the mucous coat were apparently abraded, and quite wanting at these places, without its being possible to perceive in the abraded part an ulcerated base. The mucous membrane around these portions, as on other parts of the intestine, shewed no particular appearance, except occasional dendritic vascular injections and greatly enlarged glands passing into ulceration. These greatly enlarged glands, particularly the *glandulæ solitariæ* of Brunner, might, Dr. P. observes, frequently lead people to suppose that they had found true pocks both in the small and in the large intestine; but, from a minute examination of the different forms of intestinal ulcers, he feels himself justified in expressing his conviction that those who have described small-pox as occurring in these situations had deceived themselves, and taken for small-pox pustules things of an entirely different nature.

*Nasal Cavities.* The results of Dr. P.'s examinations of these cavities he himself considers very inconclusive; for sometimes they were rendered imperfect, in consequence of his not being allowed to disfigure the body; and, in other instances, when there was nothing to prevent his doing so, the nasal canals were so filled up with mucus and pus hardened into crusts, that it was impossible to acquire an accurate knowledge of the state of the epithelium in the different stages of the disease, it being always found destroyed. The proper mucous coat frequently exhibited, after it had been cleared of all pus and mucus, ulcerated parts, of different breadth, depth, and form.

*The Larynx and Trachea, with its Branches.* Dr. P. is disposed to believe that he has found the mucous membrane of these parts more frequently affected in small-pox than that of any other organs. During the life of the patient, indeed, one might have suspected considerable alterations in the mucous membrane of the respiratory organs, from the great dyspnœa that occurred. Accordingly he always found it much inflamed, and the extremely tender epithelium, (which is here, however, much firmer than perhaps in any other situation,) could be drawn off with little trouble, although sometimes only in fragments. As in the state of health the connexion of the epithelium with the mucous membrane is so intimate as to have led many anatomists to entertain doubts respecting its existence, it may be inferred that the destruction of this connexion must have been effected by the interposition of some extraneous matter. At this stage of the disease, however, Dr. P. could discover in only one instance a fluid effused between the two membranes, so as to present a distinct vesication: yet he thinks that, from what appears in the further progress of the disease, we are warranted in believing that such an alteration occurs in all instances. In addition to the traces of inflammation, the surface of the epithelium was studded over, as it were, with a number of small dark or black points; the gaping orifices of the glands. In a short time the epithelium, which here resists softening longer than in other situations, becomes here and there dim and opaque, so as partially to conceal the redness of the subjacent mucous membrane. These dim spots are more or less round in form, and in general of the size of lentils; they can be seen very distinctly on a trachea, the vessels of which have been successfully filled with red pigment, as they are thereby brought more clearly into view. We can then see, too, that these different dim spots are not all alike; for some allow the subjacent mucous membrane to shine through, in a greater or less degree, and others not at all. If the finger be drawn over them, they are felt to be prominent; which, indeed, might be inferred, as regards the larger of them, from the shadow which they cast. These dim spots are produced by the exudation of a fluid, which begins to be effused at the time when the epithelium, as above shewn, separates from the mucous membrane. By the turbid degeneration of this fluid, and its more rapid and copious effusion, the epithelium is still more raised; and the opaque white layer of mucous or puriform matter, forming between the two membranes, prevents the mucous coat from being any longer seen, as previously, through the epithelium. That this matter really lies beneath the epithelium, is proved by the circumstances that it cannot be washed off, and that, when the light is thrown properly on the portion under examination, it is reflected equally from the whole surface, so that the epithelium, which, in consequence of its greater degree of firmness, does not begin to soften till a later period, appears everywhere equally shining and regular: and that this substance is not produced by thickening of the epithelium is shewn by the fact that the last-named membrane can be stripped off it, of the same thickness as on other parts of the mucous membrane not so affected, but merely inflamed.

In a still more advanced period there occurs softening, and the consequent destruction, of the epithelium, bringing into view a very considerable growth and expansion of these little heaps of pus, so to speak, and their frequent coalescence, till at length the whole is changed into a homogeneous, mucous, and frequently a discoloured mass, which lines the air-passages, and below which, on washing it away, is found the mucous coat much inflamed, and here and there ulcerated. Frequently, however, Dr. P. found the disease of the mucous membrane limited to particular distinct spots, so that the appearances described were perceptible only at scattered points, and ulcerated parts were seen surrounded by parts nearly in a healthy state, just as on the external skin; with this difference, that the epithelium of the air-passages could be easily separated even from the parts which were not affected. These distinct ulcers sometimes occurred in considerable number, being in some instances superficial, in others going deeper, and affecting likewise the submucous cellular texture. In all cases the glands were pretty constantly observed to be distended. Dr. P. has not seen any instance of complete perforation of the whole coats, or of ulceration extending to the cartilages. Whether appearances of the kind that have been described occurred also in the minute ramifications of the bronchi, he cannot determine, but he has certainly traced them into the third series of branches.

*Mucous Membrane of the Genital Organs.* The results derived from an examination of the mucous membrane covering both the male and the female organs of generation, were very inconsiderable; for, with the exception of the larger and smaller labia and the entrance of the vagina, in the female sex, and the inner surface of the prepuce and external surface of the glans, when not covered by the prepuce, in the male, Dr. P. found nothing remarkable. The small-pox pustules existing in these situations seemed to observe quite the same course as on the external skin; only they ran through their stages quicker, became earlier ripe, or did not reach the state of maturation at all, in consequence of their flattening before they had become filled with perfect pus, particularly on the nymphæ and the entrance of the vagina. In no instance could Dr. P. find inflammation on the mucous membrane of the urethra and vagina; but he several times found increased secretion of mucus, with great swelling of the glands of these parts.

*The Serous Membranes.* The only morbid appearance which was found in repeated examinations of the *arachnoid* was a slight collection of turbid fluid between it and the dura mater, and here and there the effusion of a mucous, gelatiniform matter, or plastic lymph, upon its surface; but these did not differ in any respect from what is found in the same situation after other diseases. The vessels lying under the arachnoid were very turgid, and everything indicated the state of congestion, or even of inflammation. In the same way, the *pleura* and the *pericardium* presented no peculiar pathological appearances: at least, the effusion of plastic lymph upon the pulmonary and costal pleura exhibited the same appearances which Dr. P. has himself repeatedly seen after pleurisy, or has found described and delineated by others. The *peritoneum*, however, presented some unusual appearances; for though, as in the case of the serous membranes of the head and chest, the serous covering of the intestinal canal exhibited nothing but what is usually remarked after inflammation of the intestines, (viz. the effusion of plastic lymph, and numerous adhesions of different parts of the intestine to one another or to the serous lining of the abdominal parietes, with frequently more or less effusion of serous or viscid fluid,) the part of the peritoneum covering the liver and the spleen presented appearances which Dr. P. thinks himself justified in regarding as the product of the small-pox; inasmuch as he has never himself seen anything similar in other diseases, nor found it described in others, notwithstanding the trouble which he has taken for this purpose. The appearances alluded to, and which presented themselves to Dr. P. in four instances, were the following:—The surface of the liver, as well as of the spleen, was found studded with white spots of different sizes and forms. The size varied between that of a lentil and that of a penny. The form appeared constantly circular, or somewhat oval, surrounded with not sharply defined, but rather slightly rounded, margins. In some instances, two or three neighbouring spots had run together, so as necessarily to produce a small difference of form; the cause of which, however, was easily discernible. If the point of the finger was drawn over these spots, it was distinctly perceived that they projected somewhat, and, when they were scraped pretty firmly with the handle of a scalpel, a tender membrane separated from them. This, however, tore off at the circumference of the spots, at the place where the parenchyma of the subjacent organs, which could not be seen through the spots in consequence of their opacity, became visible. In a few instances only did a small portion of membrane separate in continuity with the portions described from the parts which were unaffected. With a pair of pincers the thin membrane could be separated from all the spots continuously; and, under a compound microscope, it shewed nothing but a homogeneous mass, which reflected the light in such a way as to appear granular. No trace of blood-vessels or lymphatics, nor of globules, laminæ, or cells, could be discovered in it. On an accurate examination of these spots, after the outer membrane had been stripped off, nothing observable could be washed off from them, even by the aid of a sponge or the handle of a knife: they appeared like portions of the cellular texture filled with a white matter.

We have now given a pretty full view of this interesting and controverted subject, as given in the work of Dr. Petzholdt. We shall terminate our notice by transcribing the summary which he has given of the principal results deducible from his investigations of the morbid appearances found in the bodies of persons dying of small-pox.

1st. That there occurs softening of the undermost layers of the cuticle, and, at a later period, destruction of the connexion with the cutis.

2d. That orifices are never observable in the cuticle covering pocks.

3d. That what is called the pit, or umbilicus, is produced by the retention of the cuticle by the excretory ducts of the cutaneous glands.

4th. That all pocks have not a pit or umbilicus.

5th. That the pit, or umbilicus, never exists in pocks seated on the palm of the hand or sole of the foot.

6th. That, on the base of the pock, and consequently on the surface of the cutis, the orifice of the gland can be seen, except in the palm of the hand or sole of the foot.

7th. That the portion of the surface of the cutis not covered with pocks is also in a morbid condition.

8th. That the vessels at the base of the pock exhibit marks of inflammation; those at its circumference marks of congestion only.

9th. That the so-called "wind-pocks" are by no means empty.

10th. That the cutaneous glands are generally swollen in small-pox.

11th. That the excretory ducts of the glands, when destroyed, as well as the hairs that fall out, are regenerated.

12th. That the mucous membranes of persons affected with small-pox often exhibit inflammation, with the formation of vesicles and ulcers.

13th. That vesicles and superficial excoriation are met with on the tongue.

14th. That on the pharynx and back part of the mouth there are never pocks, but the glands there seated are much distended, and have very wide orifices.

15th. That there occurs softening of the epithelium of the œsophagus, rising into pustules.

16th. That, at a later period, the epithelium is completely destroyed.

17th. That ulcers are met with in the œsophagus, but never with *perforation* of the proper mucous coat.

18th. That no pocks are found in the stomach and intestinal canal; but there occurs destruction of the papillæ of the mucous membrane, particularly in the small intestine.

19th. That there is frequently dendritic vascular injection of the mucous membrane of the alimentary canal.

20th. That ulcerated parts are occasionally found in the mucous membrane of the nasal fossæ.

21st. That vesicles form in the trachea;

22d. And ulcers occur in both larynx and trachea.

23d. That the mucous membrane of the genital organs is free from pocks, except at its junctions with the external skin.

24th. That on the serous membranes nothing more is, in general, to be seen than what is found after their inflammation in other diseases.

25th. That, in a few cases, an eruption was found on the serous coverings of the spleen and liver.

---

